# Assessment of the HNF1B Score as a Tool to Select Patients for ***HNF1B*** Genetic Testing

**DOI:** 10.1159/000398819

**Published:** 2015-05-22

**Authors:** Rhian Clissold, Beverley Shields, Sian Ellard, Andrew Hattersley, Coralie Bingham

**Affiliations:** ^a^Institute of Biomedical and Clinical Science, University of Exeter Medical School, Exeter, UK; ^b^NIHR Exeter Clinical Research Facility, University of Exeter Medical School, Exeter, UK; ^c^Renal Unit, Royal Devon and Exeter Hospital, Exeter, UK

**Keywords:** Area under the curve, Genetic diseases, Kidney disease

## Abstract

**Background/Aims:**

Diagnosing hepatocyte nuclear factor 1β (HNF1B)-related disease is a challenging task due to the phenotypic variability and frequent absence of a family history. An HNF1B score has recently been developed to help select appropriate patients for genetic testing with a negative predictive value (NPV) of 99%. We aimed at testing the clinical utility of this score in a large number of referrals for *HNF1B* genetic testing to the UK diagnostic testing service for the *HNF1B* gene.

**Methods:**

An HNF1B score was assigned for 686 UK referrals for *HNF1B* genetic testing using clinical information available at referral. The performance of the score was evaluated by receiver-operating characteristic curve analysis. The relative discriminatory ability of different clinical features for making a genetic diagnosis of HNF1B-related disease were estimated in the UK dataset alone and pooled with French data.

**Results:**

The HNF1B score discriminated between patients with and without a mutation reasonably well with an area under the curve of 0.72. Applying the suggested cut-off score of ≥8 gave a NPV of 85%. In a pooled analysis, antenatal renal abnormalities, renal hyperechogenicity and cysts were discriminatory in children, whereas renal hypoplasia and cysts were discriminatory in adults. Pancreatic abnormalities were discriminatory in both, whereas other extra-renal characteristics had a large effect size only in adults.

**Conclusion:**

The HNF1B score was discriminatory for *HNF1B* mutations in a large cohort of individuals tested in a single UK centre. The lower NPV (85 vs. 99%) reduces its clinical utility in selecting patients for *HNF1B* genetic testing, although validation in a prospective cohort is required.

## Introduction

Heterozygous mutations in the gene encoding the transcription factor hepatocyte nuclear factor 1β (HNF1B) result in a multi-system disorder. They are the most commonly known monogenic cause of developmental kidney disease, which is present in the majority of cases [1,2,3]. The renal phenotype is very variable; cysts are the most frequent feature but single kidneys, hypoplasia, horseshoe kidneys, duplex kidneys, collecting system abnormalities, bilateral hydronephrosis and hyperuricaemic nephropathy may also be seen [1,4,5,6,7,8,9,10]. HNF1B-related disease is often detected on prenatal ultrasound scanning, where bilateral hyperechogenic kidneys with normal or slightly increased size are commonly found [11]. Other clinical features include young-onset diabetes, pancreatic hypoplasia, genital tract malformations, deranged liver function tests, hypomagnesaemia, hyperuricaemia and early-onset gout [9,10,12,13,14,15,16,17]. Genetic changes comprise whole-gene deletions in approximately 50% of patients and base substitutions or small insertions-deletions in the remainder [7,18]. Both may arise spontaneously; *de novo* whole-gene deletions are seen in about 50% of cases [6,11,19]. This means there is often no family history of renal disease or diabetes.

Given the marked clinical heterogeneity of HNF1B-related disease and frequent absence of a relevant family history, diagnosis is often challenging and it is likely that many cases remain undetected. Faguer and colleagues have recently developed a HNF1B score as a tool to help healthcare professionals select appropriate patients for genetic testing (table 1) [20]. It is calculated using 17 items, which include family history, antenatal discovery and organ involvement. The score performed well when tested in a cohort of 433 patients referred to the University Hospital of Toulouse in France for *HNF1B* gene analysis, with a negative predictive value (NPV) >99% and sensitivity of 98.2% using a cut-off score of 8. We aimed at replicating this study by testing the clinical utility of the HNF1B score in a cohort of 686 patients who had undergone genetic testing for *HNF1B* mutations at Exeter Molecular Genetics Laboratory, which provides the UK national diagnostic testing service for the *HNF1B* gene.

## Concise Methods

Probands with renal disease referred for *HNF1B* genetic testing to Exeter Molecular Genetics Laboratory from 1998 to 2012 were included; the criterion for referral was suspicion of HNF1B-related disease by the referring clinician. Informed consent was obtained from individuals to perform genetic testing as part of their clinical care and the study was conducted in agreement with the Declaration of Helsinki Principles. Mutation screening was performed by sequencing of coding exons and exon-intron boundaries together with gene dosage assessment by multiplex ligation-dependent probe amplification as previously described [7,19]. Clinical details were obtained from referral information and used to assign an HNF1B score as described by Faguer et al. [20].

The characteristics of interest were renal structural anomalies not due to other recognised causes (including antenatal renal abnormalities, hyperechogenicity, cysts, hypoplasia, multicystic dysplastic kidney, urinary tract malformations, solitary kidney and glomerular cysts/oligomeganephronia on biopsy), young-onset diabetes (defined by age at diagnosis ≤35 years), pancreatic hypoplasia or evidence of pancreatic exocrine failure (either reduced faecal elastase or requirement for enzyme replacement therapy), a positive family history of either renal disease or diabetes in parent/child, genital tract malformations (including aplasia of the uterus and upper vagina, bicornuate uterus, hemiuterus, absence of vas deferens and epididymal cysts), liver test abnormalities of unknown aetiology, hypomagnesaemia (serum Mg^2+^ <0.7 mmol/l) and early-onset gout (defined by age at diagnosis <30 years).

The discriminatory ability of clinical features was determined by comparing proportions in patients with and without an *HNF1B* gene mutation. Pooled odds ratios (OR) were estimated for different characteristics using both this UK dataset and published data in the recent paper by Faguer et al. from a cohort of 433 patients referred to the University Hospital of Toulouse in France for *HNF1B* gene analysis [20].

Performance of the HNF1B score in the UK cohort was evaluated by receiver-operating characteristic (ROC) curve analysis. NPV, positive predictive value (PPV), sensitivity and specificity were calculated using the recommended cut-off score of 8.

### Statistical Analyses

Differences in the frequencies of clinical features and HNF1B score were assessed using the Fisher's exact test for categorical variables and the Mann-Whitney U test for continuous variables. Effect size estimates were summarised using OR with 95% confidence intervals (CI). Pooled OR were estimated using the Mantel-Haenszel method with a calculation of 95% CI using the Robins, Breslow and Greenland variance formula. Discrimination between patients with and without an *HNF1B* mutation was assessed by determining the area under the curve of the ROC curve derived from the score. A p value of <0.05 was considered to be statistically significant. All analyses were carried out using SPSS (version 22) and StatsDirect (version 2.7.8) statistical software.

## Results

### UK Cohort Description

The cohort included 686 unrelated patients, with a male:female ratio of 1:1. Four hundred and sixteen individuals (60.6%) were aged ≤16. The majority of the cohort had a congenital anomaly of the kidney or urinary tract (CAKUT): 408 children (98.1%) and 246 adults (91.1%). A total of 177 patients (25.8%) from the 686 referred for genetic testing were found to have a heterozygous *HNF1B* gene anomaly: 78 (44.1%) had base substitutions or small insertions-deletions, 92 (52.0%) had whole-gene deletions and 7 had partial-gene deletions (4.0%).

The characteristics of the cohort are summarised in table 2. In the paediatric population, detection of antenatal renal abnormalities, renal hyperechogenicity and renal cysts were all more common in *HNF1B* mutation carriers (p = 0.0003, p = 0.0008 and p = 0.001, respectively). This is in contrast to the adult population where hypoplasia was the only discriminatory renal characteristic (p < 0.0001). Young-onset diabetes was the only clinical feature to discriminate between patients with and without an *HNF1B* mutation in both the paediatric and adult cohorts (p = 0.0002 and p < 0.0001, respectively). The median age at diagnosis of diabetes was also lower in those with HNF1B-related disease at 16.5 years (IQR 12-26.8) compared to those without (median age 32.5, IQR 15.3-49.8), p < 0.0001. Pancreatic hypoplasia and/or exocrine failure was significantly associated with *HNF1B* mutations in adults (p = 0.0006) but not children; however, there was only one affected patient in the paediatric cohort and so the numbers were too small to draw any conclusions. In the paediatric population, the frequency of other clinical features and a positive family history of renal disease or diabetes did not vary between patients with and without a diagnosis of HNF1B-related disease. However, in the adult population genital tract malformations, liver test abnormalities and hypomagnesaemia were all discriminatory (p = 0.01, p < 0.0001 and p < 0.0001, respectively).

### Estimation of Effect Size in UK and French Cohorts

**Fig. 1:**
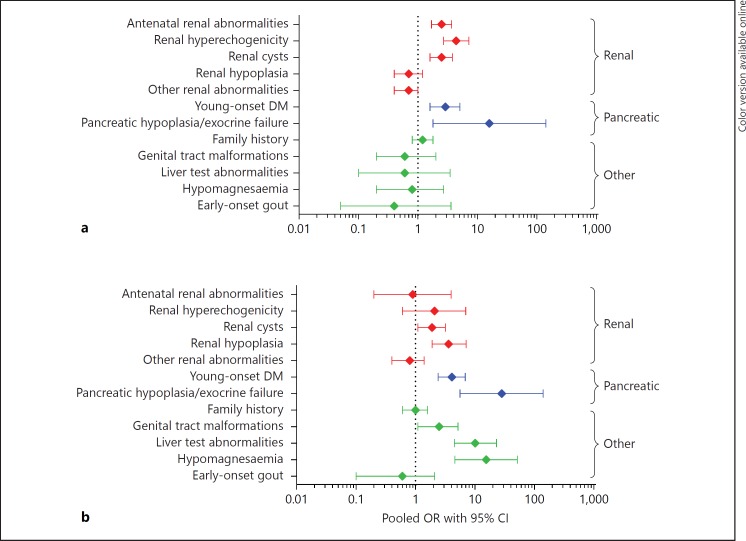
Forest plot showing the pooled OR for making a genetic diagnosis of HNF1B-related disease for different clinical features in the paediatric (**a**) and the adult cohorts (**b**) of the combined referrals for *HNF1B* genetic testing to both Exeter, UK and Toulouse, France (n = 1,119). Other renal abnormalities include multicystic dysplastic kidney, urinary tract malformations, single kidney and glomerular cysts/oligomeganephronia on biopsy. CI = Confidence interval; DM = diabetes mellitus; HNF1B = hepatocyte nuclear factor 1β; OR = odds ratio.

We then used pooled data from UK and French cohorts to see if the same clinical features were discriminatory; pooled OR were used to estimate effect size for the different characteristics (fig. 1). In the paediatric referrals, antenatal renal abnormalities, renal hyperechogenicity and cysts were the renal characteristics with the largest OR of 2.5 (95% CI 1.7-3.7), 4.4 (95% CI 2.7-7.2) and 2.5 (95% CI 1.6-3.8), respectively. OR for young-onset diabetes and pancreatic hypoplasia/exocrine failure were 2.9 (95% CI 1.6-5.1) and 15.9 (95% CI 1.8-143). In the adult referrals, hypoplasia and cysts were the renal structural anomalies with the highest OR (3.6, 95% CI 1.9-7.1, and 1.9, 95% CI 1.1-3.2, respectively). OR for the pancreatic phenotype were similar to those seen in children. Large OR were seen for genital tract malformations (2.5, 95% CI 1.1-5.2), liver test abnormalities (10.1, 95% CI 4.5-23) and hypomagnesaemia (15.5, 95% CI 4.6-52). This is in contrast to the paediatric population where the OR for these other clinical features were all <1. The 95% CI for some of these characteristics, such as pancreatic hypoplasia or exocrine failure, are very wide and this reflects the small number of patients affected.

### Evaluation of HNF1B Score in UK Cohort

**Fig. 2:**
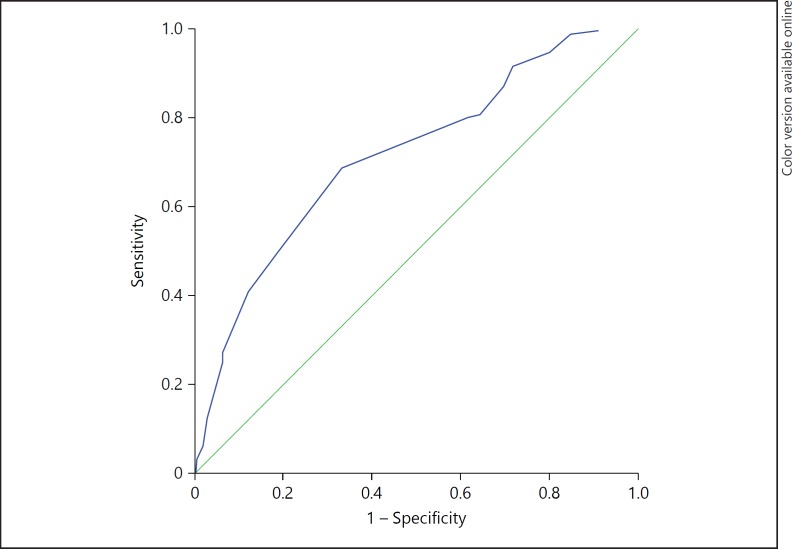
ROC curve showing the discriminative ability of the *HNF1B* score for all referrals for *HNF1B* genetic testing to Exeter Molecular Genetics Laboratory. c-statistic = 0.72 (95% CI 0.67-0.76). c-statistic was 0.71 (95% CI 0.65-0.76) in the paediatric cohort and 0.75 (95% CI 0.69-0.82) in the adult cohort. CI = Confidence interval.

The median HNF1B score was higher in patients with an *HNF1B* mutation as compared with those without (10 (IQR 8-13.5) vs. 8 (IQR 4-10), p < 0.0001). There was no significant difference in score between those with whole-gene deletions and those with base substitutions or small insertions-deletions. The ROC curve, with *HNF1B* genetic test result as the dependent variable, is shown in figure 2; area under the curve = 0.72 (95% CI 0.67-0.76). Using the suggested cut-off score of 8 gave a sensitivity of 80%, specificity of 38%, NPV of 8% and PPV of 31%. The statistical performance of the HNF1B score using different cut-off scores is shown in online supplementary table S1 (for all online suppl. material, see www.karger.com/doi/​10.1159/000398819).

## Discussion

In this study, we retrospectively generated an HNF1B score for 686 referrals for *HNF1B* genetic testing to one UK centre and found it discriminated between patients with and without a mutation reasonably well with an area under the curve of 0.72. This provides further evidence that this clinical scoring system may be a useful screening tool to select individuals for *HNF1B* genetic testing. Applying the suggested cut-off score of 8 gave a sensitivity of 80% and an NPV of 85%, so this threshold cannot be reliably used to exclude individuals with a lower score from genetic testing.

This work had limitations, which may explain the lower NPV of the HNF1B score in this cohort (85 vs. 99.4% in the original French cohort). Thirty five confirmed *HNF1B* mutation carriers in our referrals had a score below 8, and so they would not have been initially considered for *HNF1B* genetic testing according to the diagnostic strategy suggested by Faguer and colleagues [20]. Some of these false negative results may be the result of the score being calculated retrospectively using clinical details available at the time of referral. These were based on routinely collected clinical information so not all characteristics were systematically assessed for. We also included all patients who underwent *HNF1B* genetic testing at our centre from 1998 to 2012 and some of the clinical features, such as hypomagnesaemia, have only been associated with HNF1B-related disease in recent years. Use of the HNF1B score is suggested as part of a diagnostic algorithm where genetic testing should be reconsidered in individuals with a score <8 if new features suggestive of HNF1B-related disease occur. Many of the patients in our dataset may have scored ≥8 with more complete data during follow-up. It will therefore be important to assess the performance of the score in a prospective study.

There are differences between the UK and French datasets. In the UK cohort, many of the renal structural anomalies were less common in individuals regardless of their *HNF1B* status compared to a large group of patients referred to a centre in France for *HNF1B* gene analysis; antenatal renal abnormalities were seen in 137/686 (20.0%) UK referrals but 153/433 (35.3%) French referrals [20]. In contrast, young-onset diabetes was more prevalent in the UK cohort with 33.9% of *HNF1B* mutation carriers affected compared to only 5.4% in the French dataset. These differences are likely to reflect the fact that the Exeter Molecular Genetics Laboratory has a particular interest in maturity-onset diabetes of the young (MODY), whereas the University Hospital of Toulouse specialises in inherited renal disease. However, it also highlights the importance of appropriate counselling and monitoring for diabetes in affected individuals and their families. In both UK and French datasets, patients underwent genetic testing based on clinician suspicion of HNF1B-related renal disease and the majority had CAKUT. This has led to a selection bias that limits the applicability of the study results. This is in keeping with the literature to date, where the majority of cohorts with HNF1B-related disease that have been described were pre-selected for particular kidney abnormalities [1,2,3,4,6,7,9,11,19].

In the absence of any current population-based data, these two large datasets provide an important source of information on HNF1B-related disease. Similar clinical features discriminated between patients with and without an *HNF1B* mutation in both UK and pooled datasets, with both showing differences between paediatric and adult cohorts. Antenatal renal abnormalities, renal hyperechogenicity and cysts were discriminatory in children, whereas renal hypoplasia and cysts were discriminatory in adults. Pancreatic abnormalities were discriminatory in both age groups whereas genital tract malformations, liver test abnormalities and hypomagnesaemia all had a large effect size in adults only. Due to the small numbers, some of the OR have very large CI so the true effect size is difficult to estimate. However, the data suggests that the antenatal detection of renal hyperechogenicity or cysts plus the presence of either diabetes or pancreatic hypoplasia in children should prompt clinicians to consider a diagnosis of HNF1B-related disease. In adults, the renal phenotype seems to be less discriminatory, so extra-renal features are worth testing for in order to decide whether genetic testing may be required. The collection of systematic data on clinical features and biomarkers in a large cohort of *HNF1B* mutation carriers unselected for phenotype will allow more accurate modelling in different age groups and may lead to the evolution of a simpler score based only on the most discriminative features.

In summary, we have replicated the discriminative power of the recently described HNF1B score in a large cohort of individuals referred for *HNF1B* genetic testing to one UK centre. The lower NPV and sensitivity using the suggested cut-off of 8 would have led to missed cases of HNF1B-related disease in this dataset. This highlights the need for validation in a prospective cohort and recalculation of the score if a new feature of HNF1B-related disease occurs.

## Disclosure Statement

The authors declare no competing interests.

Additional material

Supplementary table supplied by authors.

## Figures and Tables

**Table 1:** HNF1B score created by Faguer and colleagues [20]

Characteristics	Item	Value
Family history		+2
Antenatal renal abnormalities	Uni/bilateral abnormality by prenatal renal ultrasound scanning	+2
Kidneys and urinary tract		
Left kidney	Hyperechogenicity	+4
	Renal cysts	+4
	Hypoplasia	+2
	Multicystic and dysplastic kidney	+2
	Urinary tract malformation	+1
	Solitary kidney	+1
Right kidney	Hyperechogenicity	+4
	Renal cysts	+4
	Hypoplasia	+2
	Multicystic and dysplastic kidney	+2
	Urinary tract malformation	+1
	Solitary kidney	+1
Electrolyte or uric acid disorders	Low serum Mg^2+^ (<0.7 mmol/l)	+2
	Low serum K+ (<3.5 mmol/l)	+1
	Early-onset gout (>30 years of age)	+2
Pathological findings	Oligomeganephronia or glomerular cysts	+1
Pancreas^a^	MODY or hypoplasia of tail and neck of the pancreas or pancreatic exocrine insufficiency	+4
Genital tract	Genital tract abnormality^b^	+4
Liver	Liver test abnormalities of unknown origin^c^	+2

HNF1B = Hepatocyte nuclear factor 1β; MODY = maturity-onset diabetes of the young.

This score should be assessed after ruling out easily recognisable inherited renal diseases, e.g., autosomal dominant or recessive polycystic kidney disease and renal coloboma syndrome.

a Maximal value of the item pancreas is 4.

b Bicornuate uterus, hemiuterus, uterus and upper vagina aplasia, epididymal cysts, bilateral absence of vas deferens.

c After exclusion of autoimmune, toxic or viral hepatitis.

**Table 2:** Characteristics of 686 patients tested for a *HNF1 B* mutation at Exeter Molecular Genetics Laboratory

	*Total* HNF1B status		p OR (95% CI)	*Children (≤16 years)* HNF1B status		p OR (95% CI)	*Adults (>16 years)* HNF1B status		p OR (95% CI)
	mutation, % (n = 177)	normal, % (n = 509)		mutation, % (n = 116)	normal, % (n = 300)		mutation, % (n = 61)	normal, % (n = 209)	
*Renal phenotype*									

Antenatal renal abnormalities	54 (30.5)	83 (16.3)	**≤0.0001** 2.3 (1.5–3.4)	53 (45.7)	80 (26.7)	**0.0003** 2.3 (1.5–3.6)	1 (1.6)	3 (1.4)	1 1.1 (0.1–11.2)

Hyperechogenicity	23 (13.0)	22 (4.3)	**0.0002** 3.3 (1.8–6.1)	21 (18.1)	20 (6.7)	**0.0008** 3.1 (1.6–6.0)	2 (3.3)	2 (1.0)	0.2 3.5 (0.5–25.4)

Renal cysts	136 (76.8)	314 (61.7)	**0.0002** 2.1 (1.4–3.0)	93 (80.2)	190 (63.3)	**0.001** 2.3 (1.4–3.9)	43 (70.5)	124 (59.3)	0.1 1.6 (0.9–3.0)

Hypoplasia	21 (11.9)	24 (4.7)	**0.002** 2.7 (1.5–5.0)	6 (5.2)	16 (5.3)	1 1.0 (0.4–2.5)	15 (24.6)	8 (3.8)	**≤0.0001** 8.2 (3.3–20.5)

Multicystic and dysplastic kidney	6 (3.4)	26 (5.1)	0.4 0.7 (0.3–1.6)	6 (5.2)	25 (8.3)	0.3 0.6 (0.2–1.5)	0	1 (0.5)	1 0 (0–65.1)

Urinary tract malformations	19 (10.7)	52 (10.2)	0.9 1.1 (0.6–1.8)	13 (11.2)	29 (9.7)	0.7 1.2 (0.6–2.4)	6 (9.8)	23 (11.0)	1 0.9 (0.3–2.3)

Solitary kidney	14 (7.9)	54 (10.6)	0.4 0.7 (0.4–1.3)	6 (5.2)	21 (7)	0.7 0.7 (0.3–1.8)	8 (13.1)	33 (15.8)	1 0.8 (0.4–1.8)

Glomerular cysts or oligomeganephronia on biopsy	4 (2.3)	10 (2.0)	0.8 1.2 (0.4–3.7)	2 (1.7)	8 (2.7)	0.7 0.6 (0.1–3.1)	2 (3.3)	2 (1.0)	0.2 3.5 (0.5–25.4)

*Pancreas phenotype* Diabetes with age of onset ≤35 years	60 (33.9)	59 (11.6)	**≤0.0001** 3.9 (2.6–5.9)	26 (22.4)	25 (8.3)	**0.0002** 3.2 (1.7–5.8)	34 (55.7)	34 (16.3)	**«b>0.0001** 6.5 (3.5–12.1)

Hypoplasia or exocrine failure	7 (4.0)	1 (0.2)	**0.0004** 20.7 (2.5–169)	1 (0.9)	0	0.3 −	6 (9.8)	1 (0.5)	**0.0006** 22.7 (2.7–192)

*Other features*									

Family history	64 (36.2)	184 (36.1)	1 1.0 (0.7–1.4)	36 (31.0)	76 (25.3)	0.3 1.3 (0.8–2.1)	28 (45.9)	108 (51.7)	0.5 0.8 (0.4–1.4)

Genital tract malformations	9 (5.1)	16 (3.1)	0.2 1.7 (0.7–3.8)	1 (0.9)	8 (2.7)	0.5 0.3 (0.04–2.6)	8 (13.1)	8 (3.8)	**0.01** 3.8 (1.4–10.6)

Liver test abnormalities	15 (8.5)	5 (1.0)	**≤0.0001** 9.3 (3.3–26.1)	0	2 (0.7)	1 0 (0–9.0)	15 (24.6)	3 (1.4)	**≤0.0001** 22.4 (6.2–80.5)

Hypomagnesaemia	11 (6.2)	7 (1.4)	**0.001** 4.8 (1.8–12.5)	3 (2.6)	6 (2)	0.7 1.3 (0.3–5.3)	8 (13.1)	1 (0.5)	**≤0.0001** 31.4 (3.8–257)

Early-onset gout	3 (1.7)	13 (2.6)	0.8 0.7 (0.2–2.3)	1 (0.9)	6 (2)	0.7 0.4 (0.05–3.6)	2 (3.3)	7 (3.3)	1 1.0 (0.2–4.8)

CI = Confidence interval; HNF1B = hepatocyte nuclear factor 1β; OR = odds ratio.
