# Supplementary material for: Assessment of the HNF1B Score as a Tool to Select Patients for HNF1B Genetic Testing
Source: Nephron. 2015 May 22;130(2):134–40. doi: 10.1159/000398819 (PMC4822678; doi:10.1159/000398819)
Supplement: Supplementary file 1 — Supplementary table supplied by authors. [file 000398819_sm_Table.docx]

| Supplementary Table S1. Sensitivity, specificity, negative predictive value and positive predictive value of HNF1B score using different cut-off scores | | | | | |
| --- | --- | --- | --- | --- | --- |
| **HNF1B score** | **Sensitivity (%)** | **Specificity (%)** | **Negative predictive value (%)** | **Positive predictive value (%)** | **Missed cases (*n*)** |
| ≥2 | 100 | 7 | 100 | 27 | 0/177 |
| ≥4 | 95 | 20 | 92 | 29 | 9/177 |
| ≥5 | 92 | 28 | 91 | 31 | 15/177 |
| ≥6 | 86 | 31 | 87 | 30 | 24/177 |
| ≥7 | 81 | 36 | 84 | 30 | 34/177 |
| ***≥8*** | ***80*** | ***38*** | ***85*** | ***31*** | ***35/177*** |
| ≥9 | 70 | 65 | 86 | 41 | 54/177 |
| ≥10 | 69 | 67 | 86 | 42 | 55/177 |
| ≥12 | 41 | 88 | 81 | 54 | 104/177 |
| Abbreviations: HNF1B, hepatocyte nuclear factor 1β.  Figures for the suggested cut-off score of 8 are given in bold italics. For comparison, sensitivity was 98%, specificity 41%, negative predictive value 99% and positive predictive value 20% using a threshold of 8 in the work published by Faguer and colleagues and only 1 of the 56 confirmed cases would have been missed.[20] | | | | | |
